# Endoscopic ultrasound‐guided gastrojejunostomy versus robotic gastrojejunostomy for unresectable malignant gastric outlet obstruction

**DOI:** 10.1002/deo2.248

**Published:** 2023-05-23

**Authors:** Rishi Pawa, Nicholas J Koutlas, Greg Russell, Perry Shen, Swati Pawa

**Affiliations:** ^1^ Department of Medicine Wake Forest School of Medicine Winston‐Salem North Carolina USA; ^2^ Biostatistics and Data Science Wake Forest School of Medicine Winston‐Salem USA; ^3^ Department of Surgery Wake Forest School of Medicine Winston‐Salem North Carolina USA

**Keywords:** endosonography, gastric bypass, gastric outlet obstruction, robotic surgical procedures, stents

## Abstract

**Objectives:**

Malignant gastric outlet obstruction (GOO) has traditionally been managed with enteral stenting and surgical gastrojejunostomy. Our study aimed to compare outcomes between endoscopic ultrasound‐guided gastrojejunostomy (EUS‐GJ) using a lumen‐apposing metal stent and robotic GJ (R‐GJ) for unresectable malignant GOO.

**Methods:**

Patients undergoing EUS‐GJ or R‐GJ for unresectable malignant GOO were retrospectively analyzed. The primary outcome was clinical success defined by the ability to tolerate oral intake at the time of discharge. Secondary outcomes included technical success, procedure duration, adverse events, and post‐procedure length of stay (LOS).

**Results:**

A total of 44 patients met the inclusion criteria. Of the 44, 29 underwent EUS‐GJ and 15 underwent R‐GJ. Age, gender, malignant etiology, and presence of ascites were similar between the two groups. Patients treated with EUS‐GJ had a higher mean Charlson comorbidity index (10.3 vs. 7.0; *p ≤* 0.0001) and a lower preoperative body mass index (22.3 vs. 27.2; *p* = 0.007). Technical and clinical success was achieved in 100% of patients in both groups (*p* > 0.99). EUS‐GJ was associated with shorter procedure duration (57.5 vs. 146.3 min; *p* < 0.0001), hospital LOS (4.3 vs. 8.2 days, *p* = 0.0009), and time to oral intake (1.0 vs. 5.8 days; *p* < 0.0001) when compared to R‐GJ. Adverse events occurred in 5 of the R‐GJ patients and none of the EUS‐GJ patients (*p* = 0.003).

**Conclusions:**

EUS‐GJ has similar efficacy and superior clinical outcomes compared to R‐GJ in the management of malignant GOO. Prospective studies with longer follow‐up duration are needed to validate these findings.

## INTRODUCTION

Malignant gastric outlet obstruction (GOO) can occur as a sequela of upper gastrointestinal, pancreatobiliary, and metastatic neoplasms. It is associated with substantial morbidity and decreased quality of life due to symptoms such as abdominal pain, nausea, vomiting, and anorexia.^1^ Unfortunately, these tumors are resectable in only 15%–20% of the cases and thus carry a very poor prognosis with a median survival of 3–6 months.^2^ As such, treatment focuses on the palliation of symptoms with the restoration of nutritional status.

Traditionally, palliation of malignant GOO was accomplished with surgical gastrojejunostomy (S‐GJ). However, S‐GJ has been associated with significant morbidity (up to 55%) and mortality.^3^, ^4^ Enteral stenting (ES) of malignant GOO with self‐expandable metal stents has been used as a non‐surgical method of palliation and offers high rates of technical and clinical success.^2^ Patients treated with ES have fewer adverse events and shorter hospital length of stay (LOS), procedure duration, and time to the resumption of oral intake compared to those treated with S‐GJ.^2^, ^5^ However, higher rates of required re‐interventions have been reported.^1^, ^2^, ^4^

To combat some of the limitations of ES and S‐GJ, endoscopic ultrasound‐guided GJ (EUS‐GJ) has been developed as an additional application of lumen‐apposing metal stents (LAMS). EUS‐GJ involves the creation of a bypass around the obstructing lesion using a LAMS without subjecting patients to the high morbidity and mortality of surgery. EUS‐GJ has been shown to be a safe and effective modality for treating GOO.^6^, ^7^ Compared to ES, EUS‐GJ has higher rates of clinical success, lower rates of symptom recurrence, and fewer required re‐interventions in large part due to the reduced risk of tumor ingrowth.^1^, ^8^, ^9^ Several retrospective studies comparing EUS‐GJ to either laparoscopic or open SGJ have demonstrated similar rates of clinical success, shorter hospital LOS, and fewer adverse events.^1^, ^10^, ^11^, ^12^, ^13^

While prior studies comparing EUS‐GJ to S‐GJ have focused on laparoscopic or open approaches, studies comparing EUS‐GJ to robotic GJ (R‐GJ) are lacking. In Roux‐en‐Y gastric bypass, the robotic approach has been shown to have shorter hospital LOS, fewer adverse events (namely anastomotic leaks), and lower rates of conversion to open surgery compared to the laparoscopic approach.^14^ The aim of our study was to retrospectively compare clinical outcomes of EUS‐GJ and R‐GJ in patients with malignant GOO who were not candidates for curative resection.

## METHODS

### Study design and data collection

This was a retrospective study conducted at a tertiary care academic medical center. Patients undergoing EUS‐GJ (March 2018–November 2022) or R‐GJ (November 2016–November 2022) for management of unresectable malignant GOO were identified and stored in a secured database in accordance with our institutional review board (IRB number: 00077330). Baseline patient characteristics including age, sex, Charlson Comorbidity Index (CCI), body mass index (BMI), presence of ascites, and etiology of malignant GOO were collected. Data on procedure details, adverse events, and clinical outcomes were also recorded.

### Outcomes

The primary outcome of this study was clinical success defined by the ability to tolerate oral intake without vomiting at the time of hospital discharge. Secondary outcomes included technical success, procedure duration, time from procedure to oral intake, post‐operative LOS, and adverse events. Technical success was defined by the successful deployment of the LAMS in the correct position in the EUS‐GJ group and the successful creation of a gastrojejunal bypass in the R‐GJ group. Adverse events were recorded and graded according to the American Society of Gastrointestinal Endoscopy Lexicon criteria.^15^ GOO Scoring System (GOOSS), a validated tool for assessing GOO symptoms in response to therapy, was used to quantify clinical improvement one month after treatment (Table 1).^16^ Change in BMI one‐month post‐procedure was also evaluated.

**TABLE 1:** Gastric outlet obstruction scoring system

Type of oral intake tolerated	GOOSS score
None	0
Only liquids	1
Soft solids	2
Complete diet	3

Abbreviation: GOOSS, gastric outlet obstruction scoring system.

### Statistical analysis

Categorical variables were reported as frequencies while continuous variables were reported as means with a standard deviation. Univariate analysis of patient‐related variables and clinical outcomes was completed using Fisher's exact test for categorical variables and Wilcoxon two‐sample test for continuous variables. A Kaplan‐Meier curve was also used to compare survival after EUS‐GJ and R‐GJ and significance testing was performed using a log‐rank test. Censoring was performed on the date of the last follow‐up. Cox proportional hazards regression was used to assess the effect of independent variables on survival. A *p*‐value of < 0.05 was used to determine statistical significance.

### Procedure technique

#### Endoscopic ultrasound‐guided GJ

**FIGURE 1:**
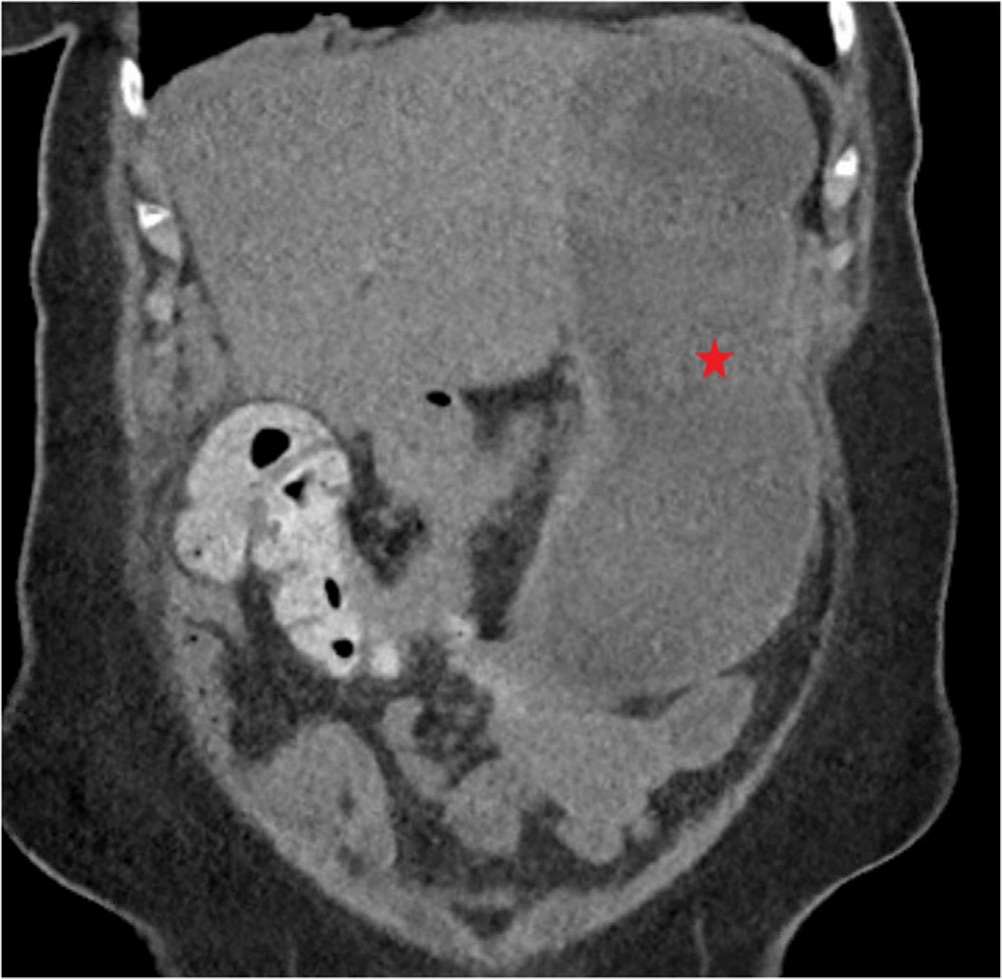
Computed tomography scan showing gastric distension (red star) with fluid and gas attributable to gastric outlet obstruction from a duodenal adenocarcinoma.

**FIGURE 2:**
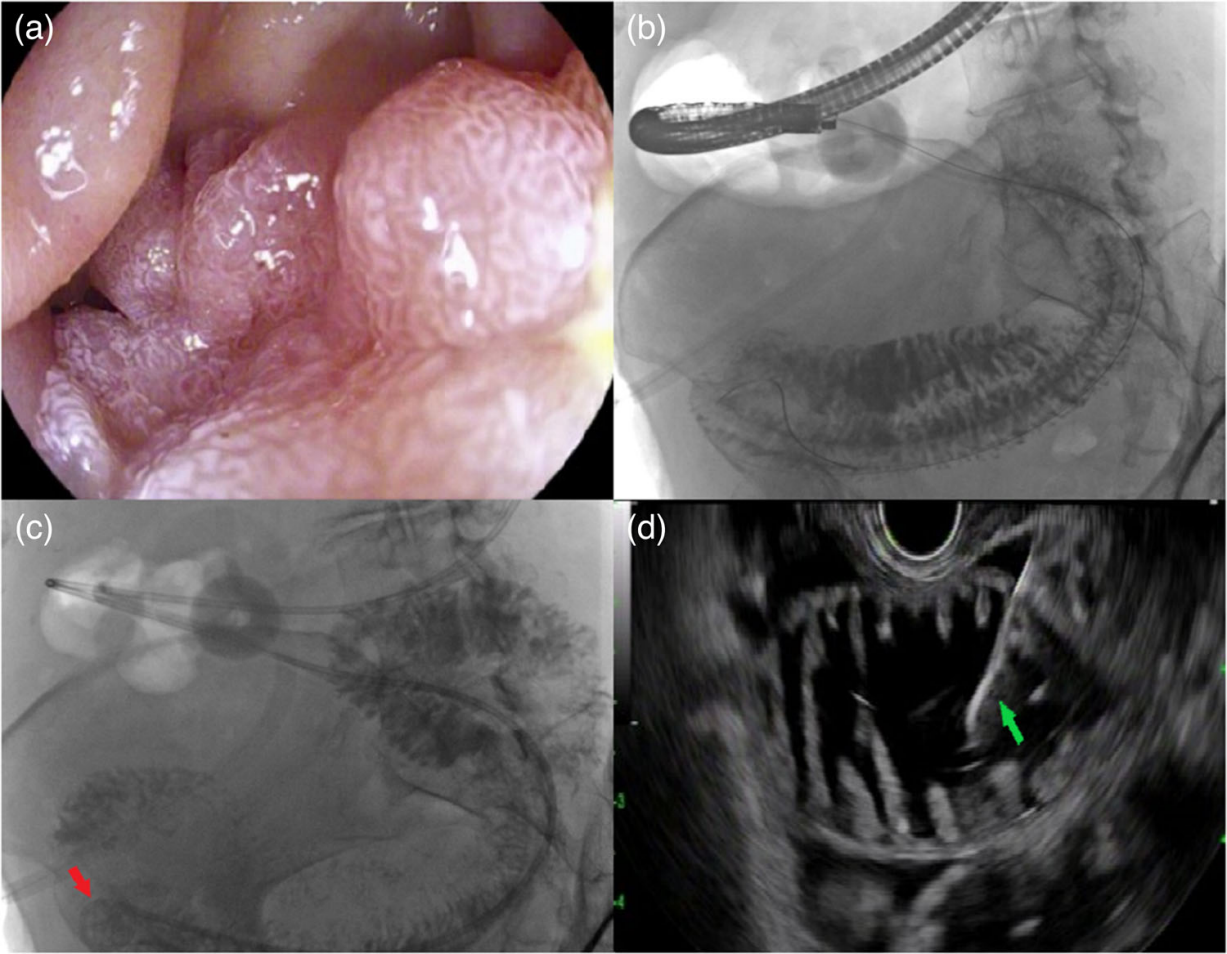
(a) Diagnostic upper endoscopy demonstrating duodenal adenocarcinoma with resultant luminal narrowing. (b) Fluoroscopic image of a Visiglide 2 wire traversing a duodenal stricture with contrast opacifying the small bowel. (c) Nasocystic catheter placement with the tip (red arrow) distal to the ligament of Treitz in the jejunum. (d) Endoscopic ultrasound image showing a distended loop of small bowel during successful fine needle aspiration of blue‐tinged fluid with a 22‐G fine needle aspiration needle.

**FIGURE 3:**
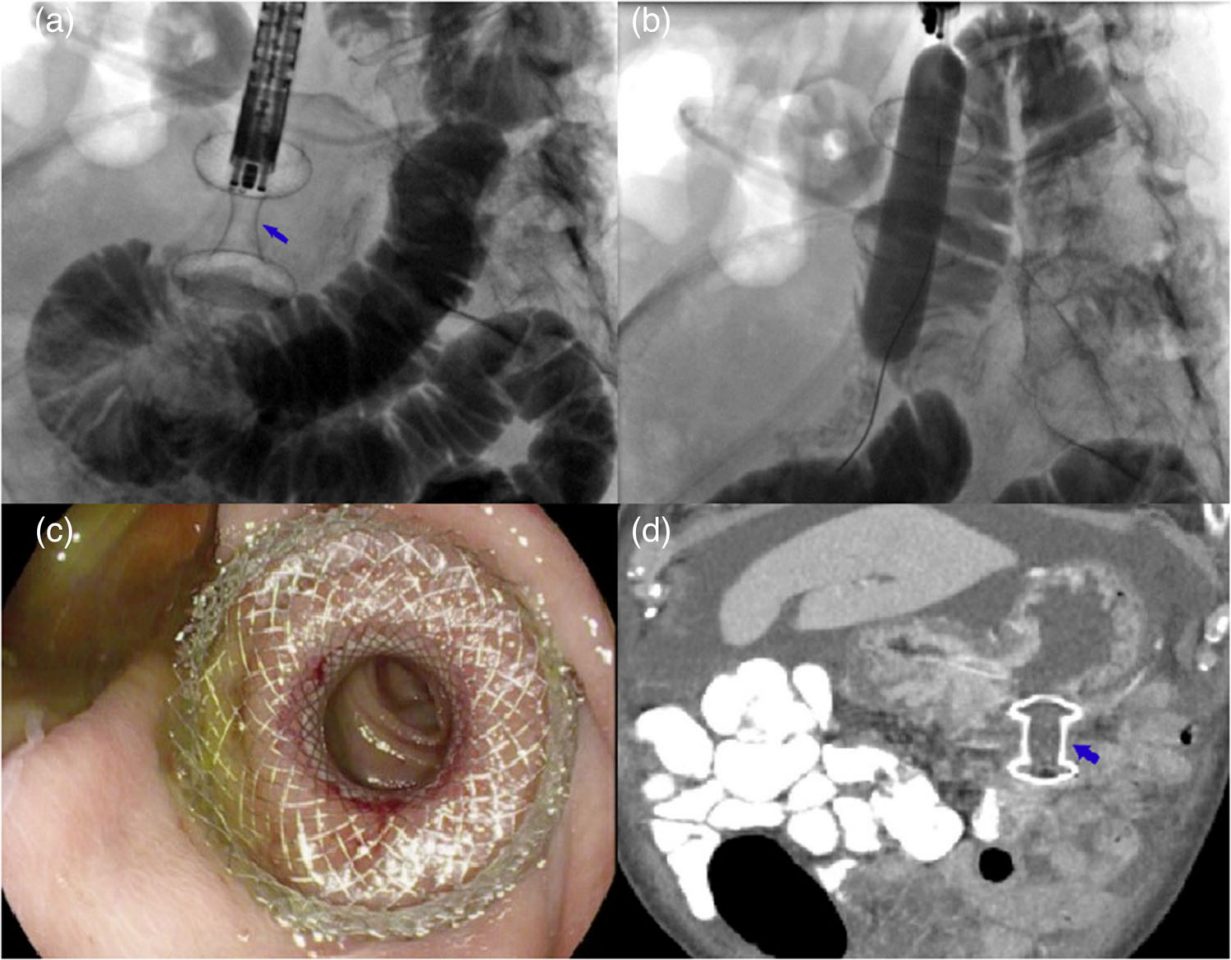
(a) Successful deployment of a 20 mm electrocautery enhanced lumen apposing metal stent (LAMS; blue arrow) with the proximal end in the stomach and distal end in the jejunum as demonstrated on fluoroscopy. (b) Balloon dilation of the LAMS to 15 mm. (c) Endoscopic image after successful creation of a gastrojejunostomy with a LAMS. (d) Computed tomography scan 1‐day post‐endoscopic ultrasound guided‐gastrojejunostomy illustrating appropriate LAMS (blue arrow) position.

All endoscopic procedures were performed by a single endoscopist (Rishi Pawa) under general anesthesia during the study period. Each patient received prophylactic antibiotics. A computed tomography scan was obtained prior to endoscopic gastrojejunostomy in all patients (Figure 1). Diagnostic esophagogastroduodenoscopy was first performed using a standard gastroscope (Pentax, Tokyo, Japan), and the site of obstruction was identified (Figure 2a). A 0.025‐inch‐diameter and 450 cm in length straight tip Visiglide 2 wire (Olympus, Center Valley, PA, USA) was advanced past the obstruction into the small bowel (Figure 2b). The proximal small bowel was opacified with contrast injection. A 7 Fr nasocystic catheter was advanced over the guidewire and positioned beyond the ligament of Treitz in the proximal jejunum (Figure 2c). Keeping the catheter in position, the gastroscope was exchanged with a linear echoendoscope (GF‐UCT180; Olympus). Dilute contrast mixed with methylene blue was infused via the catheter into the small bowel. A distended small bowel loop was identified from the stomach and punctured with a 22‐G needle (EchoTip Ultra, Cook Medical, Winston‐Salem, NC, USA; Figure 2d). Blue‐colored fluid was aspirated indicating the correct needle position in the small bowel. Subsequently, a 20 mm electrocautery enhanced lumen apposing metal stent (LAMS; Axios, Boston Scientific, Marlborough, MA, USA) was deployed to create a gastrojejunostomy using a direct technique (Figure 3a). The lumen of the stent was dilated to 15 mm following which the endoscope was advanced through the LAMS with visualization of the small bowel (Figure 3b,c). A computed tomography with oral contrast was obtained the following day to confirm the appropriate stent position after which the patient was started on a clear liquid diet and gradually advanced to a soft mechanical diet over 24 h (Figure 3d).

#### Robotic GJ

All surgical procedures were performed by a single surgeon (Perry Shen). A Veress needle was inserted into the left upper quadrant and the abdomen was insufflated using CO_2_. Three robotic ports were then placed in the left, mid, and right portions of the abdomen with 8 cm separating each port. The da Vinci Xi robot (Intuitive Surgical, Sunnyvale, CA, USA) was docked. The ligament of Treitz was subsequently identified. An area of proximal jejunum was brought up near the gastric body in an antecolic isoperistaltic fashion. Both enterotomy and gastrotomy were performed using monopolar scissors. A 12 mm assistant port was then inserted into the right upper quadrant. A 60 mm Echelon stapler (Johnson & Johnson, New Brunswick, NJ, USA) was passed through this and used to create a side‐to‐side anastomosis. The staple hole was then closed using 3‐0 monocryl Stratafix (Ethicon, Bridgewater, NJ, USA) coming from both corners of the staple line and suturing to the middle with a Connell suture, and then both ends were tied together. The robot was then undocked, all ports removed, and incisions sutured closed with 4‐0 monocryl. A nasogastric tube was placed post‐operatively in all patients and was removed once the output was less than 500 ml in 24 h. Subsequently, the patients were initiated on a clear liquid diet and advanced as tolerated. No repeat imaging was performed unless clinically indicated.

## RESULTS

### Patient characteristics

A total of 44 patients with unresectable malignant GOO treated with EUS‐GJ or R‐GJ were identified. The mean age was 65.6 (SD 12.8) years and 39% were female. Mean CCI was 9.2 (SD 2.3) and the mean pre‐operative BMI was 24.0 (SD 5.1) kg/m^2^. Pancreatic cancer was the most common etiology of malignant GOO comprising 57% of the cases. Ascites were present in 50% of the entire cohort.

Of the 44 patients, 29 underwent EUS‐GJ and 15 underwent R‐GJ. Baseline characteristics including age, gender, malignant etiology, presence of ascites, and preoperative prognostic nutritional index (PNI = 10 × albumin [g/dl] + [0.005 × lymphocytes/μl]) were equivalent in both groups (Table 2). Of note, patients undergoing EUS‐GJ had a lower preoperative BMI (22.3 vs. 27.2; *p* = 0.007) and a higher mean CCI (10.3 vs. 7.0; *p* = <0.0001) compared to the R‐GJ cohort.

**TABLE 2:** Comparison of baseline characteristics between endoscopic ultrasound guided‐gastrojejunostomy and robotic gastrojejunostomy cohorts

Characteristic	EUS‐GJ (*n* = 29)	R‐GJ (*n* = 15)	*p*‐Value
Mean age (SD), years	66.3 (12.6)	64.1 (13.7)	0.73
Female gender, *n* (%)	10 (34)	7 (47)	0.52
Pre‐operative BMI, mean (SD), kg/m^2^	22.3 (4.0)	27.2 (5.7)	0.007*
Pre‐operative PNI, mean (SD)	35.8 (6.5)	39.2 (6.5)	0.11
Charlson comorbidity index, mean (SD)	10.3 (1.8)	7.0 (1.3)	<0.0001*
Presence of ascites, *n* (%)	13 (45)	9 (60)	0.53
Malignant etiology, *n* (%)			0.72
Pancreatic cancer	15 (52)	10 (67)	
Metastatic disease	6 (21)	1 (7)	
Duodenal cancer	3 (10)	2 (13)	
Cholangiocarcinoma	2 (7)	0 (0)	
Hepatocellular carcinoma	1 (3)	0 (0)	
Gastric cancer	2 (7)	2 (13)	

* *p*‐Value < 0.05 indicates statistical significance.

Abbreviations: BMI, body mass index; EUS‐GJ, endoscopic ultrasound guided‐gastrojejunostomy; R‐GJ, robotic, gastrojejunostomy; PNI, prognostic nutritional index.

### Outcomes

Relevant outcomes are illustrated in Table 3. Technical and clinical success were similar in both groups (*p* > 0.99). The mean procedure duration was substantially longer in the R‐GJ group compared to patients undergoing EUS‐GJ (146.3 vs. 57.5 min; *p* < 0.0001). In addition, patients undergoing EUS‐GJ had a reduced time to oral intake (1.0 vs 5.8 days; *p* < 0.0001) and a shorter length of post‐operative stay (4.3 vs. 8.2 days, *p* = 0.0009) than the R‐GJ group. At the time of discharge, 93% of EUS‐GJ patients tolerated a mechanical soft diet compared to 33% of patients undergoing R‐GJ (*p* < 0.0001).

**TABLE 3:** Clinical outcomes after endoscopic ultrasound guided‐gastrojejunostomy and robotic gastrojejunostomy for patients with unresectable malignant gastric outlet obstruction

Outcome	EUS‐GJ (*n* = 29)	R‐GJ (*n* = 15)	*p*‐Value
Technical success, *n* (%)	29 (100)	15 (100)	>0.99
Clinical success, *n* (%)	29 (100)	15 (100)	>0.99
Procedure duration, mean (SD), minutes	57.5 (27.4)	146.3 (39.6)	<0.0001*
Time from GJ to oral intake, mean (SD), days	1.0 (0)	5.8 (3.8)	<0.0001*
Tolerating soft mechanical diet at discharge, *n* (%)	27 (93)	5 (33)	<0.0001*
Length of post‐operative stay, mean (SD), days	4.3 (2.7)	8.2 (4.3)	0.0009*
GOOSS score at 1 month, mean (SD)	2.9 (0.5)	2.1 (1.1)	0.010*
BMI at 1 month, mean (SD), kg/m^2^	21.5 (3.7)	24.6 (5.5)	0.095
Average BMI change, mean (SD), kg/m^2^	‐0.1 (1.7)	‐2.6 (3.5)	0.019*
Post‐operative PNI, mean (SD)	35 (6.0)	37.5 (8.5)	0.45
Average PNI change, mean (SD)	‐1.5 (5.8)	‐1.8 (7.6)	0.72

* *p*‐Value < 0.05 indicates statistical significance.

Abbreviations: BMI, body mass index; GJ, gastrojejunostomy; GOOSS, gastric outlet obstruction scoring system; PNI, prognostic nutritional index.

The average BMI change was lower in the EUS‐GJ group (EUS‐GJ vs R‐GJ, ‐0.1 [SD 1.7] vs. ‐2.6 kg/m^2^ [SD 3.5]; *p* = 0.019). Despite pre‐operative BMI being significantly lower in the EUS‐GJ cohort, there was no significant difference in post‐operative BMI at one month between the two groups (21.5 [SD 3.7] vs. 24.6 kg/m^2^ [SD 5.5]; *p* = 0.095). Post‐operative PNI at 1 month was not significantly different between the two cohorts (*p* = 0.45). At one month follow‐up, the mean GOOSS score was significantly higher in the EUS‐GJ group compared to the R‐GJ group (2.9 vs. 2.1; *p* = 0.010). There were no recurrences of gastric outlet obstruction in either cohort. One patient in the EUS‐GJ group underwent a repeat endoscopy for routine LAMS exchange one year after the index procedure to reduce the risk of stent delamination.

**FIGURE 4:**
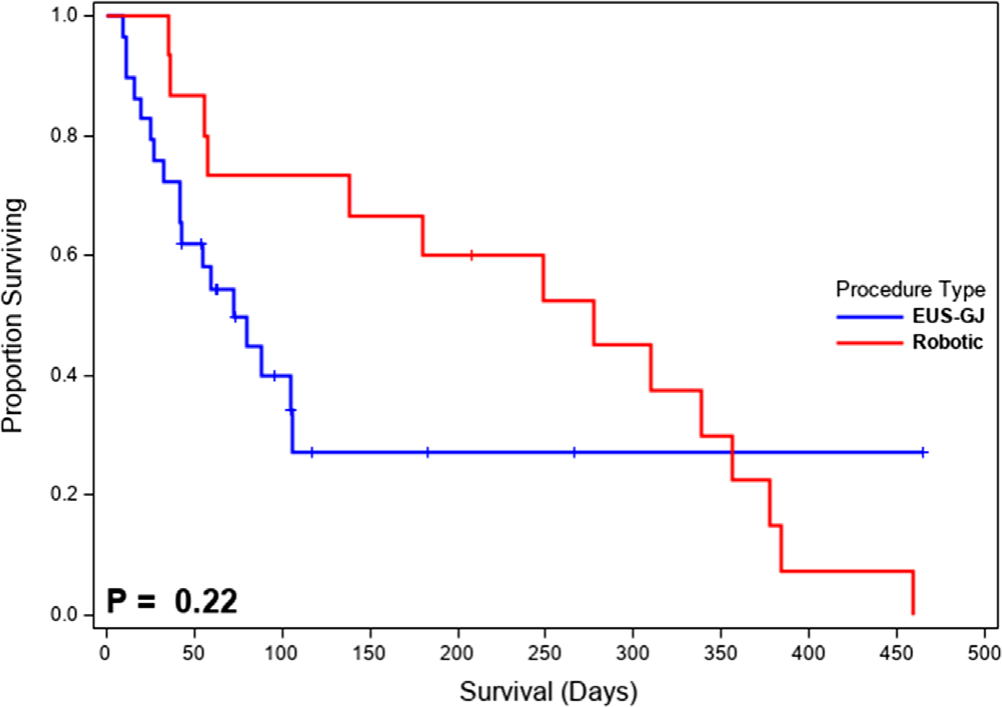
Kaplan‐Meier curve illustrating overall survival after endoscopic ultrasound guided‐gastrojejunostomy (EUS‐GJ) and robotic gastrojejunostomy (R‐GJ) for unresectable malignant gastric outlet obstruction. Overall survival was 105 days (95% confidence interval [CI] 56–277). Median survival was shorter in the EUS‐GJ cohort compared to the R‐GJ cohort (72 days [95% CI 42–106] vs. 277 days [95% CI 56–356]; *p* = 0.22).

A Kaplan‐Meier survival curve is shown in Figure 4. Overall median survival was 105 days (95% confidence interval [CI] 56–277). In comparison, the median survival was longer in the R‐GJ group as opposed to the EUS‐GJ cohort (277 [95% CI 56–356] vs. 72 days [95% CI 42–106]); however, this was not statistically significant (*p* = 0.22). In a regression model adjusting for the procedure, higher pre‐operative PNI (hazard ratio [HR] = 0.90; 95% CI 0.84–0.97; *p* = 0.006) and lower CCI (HR = 1.44; 95% CI 1.14–1.84; *p* = 0.003) were associated with improved median survival. The type of procedure (*p* = 0.19) was not associated with survival length (Table 4).

**TABLE 4:** Cox proportional hazards univariate and multivariate regression model for assessing survival

Variable	*p*‐Value	Hazard ratio	95% CI
**Univariate logistic regression model**			
Age (per 5‐year increase)	0.29	1.08	(0.94, 1.25)
Sex (female vs. male)	0.33	1.43	(0.70, 2.92)
Procedure (EUS‐GJ vs. robotic GJ)	0.22	1.60	(0.75, 3.39)
Etiology			
Pancreas versus other	0.24	0.66	(0.33, 1.32)
Pancreas versus metastatic (*n* = 32)	0.13	0.44	(0.15, 1.28)
Ascites	0.58	1.23	(0.59, 2.57)
Pre‐op BMI (per 1 unit increase)	0.47	0.98	(0.92, 1.04)
Pre‐op PNI (per 5 unit increase)	0.001	0.60	(0.43, 0.81)
CCI (per 1 unit increase)	0.001	1.37	(1.13, 1.65)

**Multivariate logistic regression model**			
Pre‐op PNI (per 1 unit increase)	0.006	0.90	(0.84, 0.97)
CCI (per 1 unit increase)	0.003	1.44	(1.14, 1.84)
Procedure (EUS‐GJ vs. Robotic GJ)	0.19	0.49	(0.17, 1.42)

### Adverse events

Adverse events related to gastrojejunostomy are summarized in Table 5. No adverse events were seen in the EUS‐GJ group. A total of five adverse events occurred in the R‐GJ cohort (33%; *p* = 0.003). Two patients developed delayed bleeding on post‐operative days (PODs) 17 and 48, respectively. One patient was managed with a blood transfusion and no active bleeding was found on endoscopy. In the second patient, active bleeding was noted from an ulcer in the afferent limb of the jejunum requiring endoscopic therapy. Sepsis developed in one patient on POD 5 requiring intensive care unit transfer. One patient had intractable nausea and vomiting requiring endoscopy on POD 5. Anastomotic narrowing secondary to edema was noted on endoscopy and managed with nasojejunal tube placement. Lastly, there was one case of a GJ volvulus that necessitated surgical detorsion with enteropexy on POD 8.

**TABLE 5:** Adverse events after endoscopic ultrasound guided‐gastrojejunostomy and robotic gastrojejunostomy

	EUS‐GJ (*n* = 29) (%)	R‐GJ (*n* = 15) (%)	*p*‐Value
Adverse events, *n* (%)	0 (0)	5 (33)	0.003
Bleeding	0	2 (13)	0.11
Sepsis	0	1 (7)	0.34
Anastomotic occlusion	0	1 (7)	0.34
GJ volvulus	0	1 (7)	0.34

* *p*‐Value < 0.05 indicates statistical significance.

Abbreviations: GJ, gastrojejunostomy; EUS‐GJ, endoscopic ultrasound guided‐gastrojejunostomy; R‐GJ, robotic, gastrojejunostomy.

Abbreviations: BMI, body mass index; CCI, Charlson Comorbidity Index; CI, confidence interval; GJ, gastrojejunostomy; EUS‐GJ, endoscopic ultrasound guided‐gastrojejunostomy; R‐GJ, robotic, gastrojejunostomy; PNI, prognostic nutritional index.

## DISCUSSION

Palliation of malignant GOO has traditionally been accomplished by S‐GJ or ES with self‐expandable metal stents. More recently, EUS‐GJ with LAMS has emerged as a viable therapy in such patients. While there are studies comparing EUS‐GJ to S‐GJ (open or laparoscopic), this is the first study to compare clinical outcomes of EUS‐GJ and R‐GJ in the management of unresectable malignant GOO.

In this study, we found similar rates of technical and clinical success in both the EUS‐GJ and R‐GJ groups. Similar clinical success was an expected result as it aligns with the results of numerous prior studies comparing EUS‐GJ and S‐GJ.^1^, ^10^, ^11^, ^12^, ^13^ In contrast, in 2017, Khashab et al. found lower rates of technical success in EUS‐GJ compared to open S‐GJ (87% vs. 100%; *p* = 0.009) in a retrospective study of 93 patients (30 EUS‐GJ, 63 S‐GJ).^10^ More recently, however, two separate retrospective studies found no difference in technical success between EUS‐GJ and S‐GJ.^12^, ^13^ The reported difference in the past was likely attributable to the learning curve of EUS‐GJ and the lack of standardized technique. Over time, further refinement in technique and increased familiarity with the procedure have led to improved technical success.

While our study found no differences in efficacy, we did note significantly shorter post‐procedure LOS in patients undergoing EUS‐GJ. This is in accordance with a recent meta‐analysis that compared EUS‐GJ (128 patients) and laparoscopic or open S‐GJ (143 patients) and found a shorter total hospital LOS for EUS‐GJ (mean difference in hospital stay between EUS‐GE and SGJ was 5.11 days; *p* < 0.01).^1^ In addition, in the EUS‐GJ group of our study, the time to resumption of oral intake was significantly shorter and the proportion of patients who tolerated a mechanical soft diet at discharge was substantially higher. These findings can likely be explained by the less invasive nature of EUS‐GJ compared to R‐GJ. Furthermore, post‐operative delayed gastric emptying after S‐GJ occurs in up to 26% of patients and may also be contributing, although data on the impact of EUS‐GJ on gastric emptying is sparse.^17^

To analyze outcomes at one month, we used the change in BMI to quantify the nutritional status and the GOOSS score to objectify symptom improvement. Despite patients in the EUS‐GJ group having lower pre‐operative BMI than R‐GJ patients, BMI one‐month post‐procedure was not significantly different between the two groups. Additionally, in the EUS‐GJ cohort, we found significantly higher GOOSS scores at one‐month post‐procedure compared to R‐GJ. These results suggest that patients treated with EUS‐GJ lose less weight than R‐GJ patients in the short term. This could be explained by faster recovery, shorter hospital LOS, and superior tolerance of an oral diet associated with EUS‐GJ. Unfortunately, long‐term analysis of these outcomes in our study was not feasible due to the high mortality and resultant small sample size.

The adverse event rate for EUS‐GJ has been reported to be lower than S‐GJ, which is congruent with our results.^11^, ^12^, ^13^ None of the patients in the EUS‐GJ group experienced adverse events while five patients in the R‐GJ cohort had complications. Based on our results, when performed by an experienced endoscopist, it appears EUS‐GJ is a safer option than any surgical modality. However, while not observed in this study, stent misdeployment is a potentially serious but not uncommon (up to 10%) adverse event that requires prompt diagnosis and management by the endoscopist, with surgical management necessary in 11% of the cases.^18^

Although not statistically significant, the median survival was longer in the R‐GJ group compared to the EUS‐GJ group (277 vs. 72 days; *p* = 0.22). Similar results were seen in a retrospective, multicenter cohort study by Khashab et al. demonstrating superior median survival in open S‐GJ compared to EUS‐GJ (148 vs. 103 days; *p* = 0.006).^10^ The findings in our study can be explained by selection bias as sicker patients may have been preferentially selected for a less invasive procedure. Furthermore, this notion is supported by a higher mean CCI (10.3 vs. 7.0; *p ≤* 0.0001) and lower mean pre‐operative BMI (22.3 vs. 27.2; *p* = 0.0070) in the EUS‐GJ group.

Our study has several notable limitations. Given its retrospective design, treatment allocation was made without randomization. As such, the study is susceptible to selection bias as described above. The small sample size in each cohort could have resulted in the study not being adequately powered to detect potential differences in clinical outcomes. In addition, these procedures were performed by an experienced endoscopist and surgeon, which may prevent the generalization of these results to proceduralists with less experience. Lastly, follow‐up regarding symptoms and nutritional status was only completed through one‐month post‐procedure making it unclear if the benefits of EUS‐GJ over R‐GJ last for more prolonged periods of time.

In conclusion, the off‐label use of EUS‐GJ for unresectable malignant GOO has a similar technical and clinical success compared to R‐GJ. In addition, shorter procedure time, reduced post‐operative LOS, fewer adverse events, and a higher GOOSS score at one month make this technique a more attractive option compared to more invasive surgical approaches. Further prospective studies with longer‐term follow‐ups are needed to confirm these findings.

## CONFLICT OF INTEREST STATEMENT

Dr Rishi Pawa is a consultant for Boston Scientific. The other authors declare no conflict of interest.
